# Assessing the Impact of Peer Educator Outreach on the Likelihood and Acceleration of Clinic Utilization among Sex Workers

**DOI:** 10.1371/journal.pone.0159656

**Published:** 2016-07-28

**Authors:** Parthasarathy Krishnamurthy, Sam K. Hui, Narayanan Shivkumar, Chandrasekhar Gowda, R. Pushpalatha

**Affiliations:** 1 Department of Marketing and Entrepreneurship, Institute for Health Care Marketing, C. T. Bauer College of Business, University of Houston, Houston, Texas, United States of America; 2 Department of Pediatrics, Baylor College of Medicine, Houston, Texas, United States of America; 3 Department of Anesthesiology, University of Texas Medical Branch, Galveston, Texas, United States of America; 4 Department of Marketing and Entrepreneurship, C. T. Bauer College of Business, University of Houston, Houston, Texas, United States of America; 5 Swasti Health Resource Center, Bangalore, Karnataka, India; 6 Swades Foundation, Mumbai, Maharashtra, India; 7 Swathi Mahila Sangha, Bangalore, Karnataka, India; Yale School of Public Health, UNITED STATES

## Abstract

**Objective:**

Peer-led outreach is a critical element of HIV and STI-reduction interventions aimed at sex workers. We study the association between peer-led outreach to sex workers and the time to utilize health facilities for timely STI syndromic-detection and treatment. Using data on the timing of peer-outreach interventions and clinic visits, we utilize an Extended Cox model to assess whether peer educator outreach intensity is associated with accelerated clinic utilization among sex workers.

**Methods:**

Our data comes from 2705 female sex workers registered into *Pragati*, a women-in-sex-work outreach program, and followed from 2008 through 2012. We analyze this data using an Extended Cox model with the density of peer educator visits in a 30-day rolling window as the key predictor, while controlling for the sex workers’ age, client volume, location of sex work, and education level. The principal outcome of interest is the timing of the first voluntary clinic utilization.

**Results:**

More frequent peer visit is associated with earlier first clinic visit (HR: 1.83, 95% CI, 1.75–1.91, *p* < .001). In addition, 18% of all syndrome-based STI detected come from clinic visits in which the sex worker reports no symptoms, underscoring the importance of inducing clinic visits in the detection of STI. Additional models to test the robustness of these findings indicate consistent beneficial effect of peer educator outreach.

**Conclusions:**

Peer outreach density is associated with increased likelihood of–and shortened duration to–clinic utilization among female sex workers, suggesting potential staff resourcing implications. Given the observational nature of our study, however, these findings should be interpreted as an association rather than as a causal relationship.

## Introduction

Peer-led outreach is a critical element of HIV and STI-reduction interventions aimed at sex workers because peers are better at contacting, connecting, empathizing, and communicating[1] with the target population. Peer-led outreach has been deployed in STI-control interventions worldwide[2, 3] including initiatives in West Bengal, India[4], Bali[5], the Philippines[6], sub-Saharan Africa[7], the National AIDS Control Program in India.

There is extensive research on the impact of these interventions[8–11] including those that examine the impact of peer-led outreach among sex workers on several impact criteria (see Table 2 in Wheeler et al^5^). These studies employ a variety of methodologies including cross-sectional surveys, field experiments and mathematical modeling^3^, and focus on a range of outcomes such as increased coverage effectiveness^6-10^ (e.g., number of sex workers registered, and condom usage), and improved knowledge about HIV and other sexually transmitted infections^3^. Recently, Pickles et al[12] assessed the impact of Avahan, a large-scale STI/HIV intervention program in India, using causal pathway analysis to analyze data from two national cross-sectional surveys called Integrated Behavioral and Biological Assessment (IBBA) surveys[13] of the vulnerable populations conducted four years apart. This study estimated that Avahan had reduced over 200,000 HIV infections. Separately, Ng and colleagues[14], using district-level program spending as an indicator for program exposure data and IBBA surveys have estimated that Avahan may have averted over 100,000 HIV infections in India. Similarly, Moses and colleagues[15] studied the impact of intensive preventive interventions targeted at sex workers on the standardized HIV prevalence rate among ante-natal clinic visitors, and found that the districts that received the intervention had a higher decline in the prevalence of HIV (56%) compared to districts that did not receive the intervention (5%). However, subsequent mathematical modeling suggests that these reductions, while substantial, cannot be attributed completely to the preventive interventions alone[16]; other explanatory factors for the decline include potential non-random selection of high-prevalence districts in the analyses reported earlier, variations in HIV trends in the ante-natal clinics studied, and variations in changes in condom use from the time the study began.

While there is some evidence that large scale social interventions are associated with demonstrable reductions in the focal illness, it remains unclear *which* of the program elements make an impact. Given that peer-led outreach has been a major component of most recent social interventions including Avahan, we believe it is important to assess whether peer-led outreach is associated with a difference to the vulnerable population of sex workers. Therefore, the first key attribute of our study is that we focus on peer-led outreach as the explanatory variable.

Second, it is well documented that the transmission of STI in a given community depends on the duration of infectivity at the individual-level[17], which in turn is influenced by how quickly the STIs are detected and treated (even if syndromically). Thus, one of the key goals of peer-led outreach is to encourage utilization of clinic services by sex workers. Specifically, peer-led outreach aims to educate the sex worker to about STI-related symptoms, and promote clinic attendance for regular check-ups and treatment of STI-related symptoms. To the best of our knowledge, the impact of interventional intensity on duration to utilization of clinic services for syndromic detection and treatment has not been studied. Thus, the second key attribute of our research is that we focus not only on whether peer-outreach density influences whether a clinic visit happens, but also the duration it takes from initial peer outreach contact to the first voluntary visit to the clinic. Further, since the key reason for encouraging clinic visits is that the clinic staff may identify STI symptoms that may be missed by the sex worker, as a secondary aim, we examine the correspondence between sex worker’s self-reported STI status and that of the STI-syndrome detected by clinic staff during the clinic visit.

To summarize, the goal of this paper is to study the association between peer-led outreach on the duration to utilize clinic services. Studying the impact on duration to clinic utilization requires individual-level information systems that track interventional intensity, service uptake and health status. To this end, we took advantage the extensive information infrastructure built into Avahan that collects time-stamped, individual-level outreach and clinic utilization metrics[18, 19] to address our research question (see Chandrasekharan et al[20] for a detailed description of Avahan evaluation design). Specifically, we combined the peer-outreach data with clinic visit data based on the sex worker identification number (common to both datasets) to create an individual-level longitudinal dataset of the timeline of peer educator outreach and clinic visits for a large sample of sex workers. The data came from four zones in Bengaluru, Karnataka, India. To the best of our knowledge, this is the first paper that that combines clinic and outreach efforts into a single dataset[21, 22] to explore the effectiveness of peer-led outreach efforts.

Like many HIV/STI prevention efforts, Avahan is a public-health intervention that takes the best knowledge from epidemiological/medical research and combines it with marketing principles of segmentation, targeting, and social influence[23, 24]. There are remarkable parallels to the influence structure in Avahan to what occurs in the domain of marketing, specifically salesforce as an interventional mechanism. We therefore drew inspiration from the literature on the impact of salesforce effectiveness on customer response[25, 26] to study the effect of peer-led outreach on clinic-utilization behavior.

## Methods

### Ethics Statement

The protocol received ethics approval by the Committee for the Protection of Human Subjects from the University of Houston.

### Study Context and Sample, Data Sources, and Key Variables

The context of the study was Project *Pragati*, an interventional program that aims to encourage sex workers to utilize clinic services using peer educator outreach as its chief intervention. *Pragati* is a joint effort between Swathi Mahila Sangha, a sex worker collective and Swasti, a not-for-profit health resource center, located in Bengaluru, India, and funded initially under the Avahan initiative of the Bill and Melinda Gates Foundation (please see monograph of Avahan[23] for a detailed description of the project).

The sample for our analysis consisted of 2705 adult (age 18 to 60) female sex workers who regularly conduct sex work at brothel, street, lodge, or home, and who were registered into Project Pragati on or after Jan 1, 2008. As discussed earlier, each sex worker was met by a peer educator in the field and was subsequently registered into the project at the health clinic. In this paper, we restricted our analysis to female sex workers whose first visit was a clinic visit, and did not receive a positive diagnosis of sexually transmitted infections during the initial clinic visit when they were registered into the project. We also excluded sex workers who did not disclose their client volume (a control variable in our analysis). This resulted in a final sample of 2705 female sex workers. For each sex worker, by matching the outreach and clinic-visit datasets, we generated a timeline that included the timing of every recorded peer educator visit, as well as that of clinic visits (if any), from the day she was registered into the program until Jan 31, 2012 (the end of the observation). In addition, for each clinic visit, the sex worker’s self-reported STI status and the STI-status based on syndromic detection by clinic staff is known. This allowed us to test whether clinic visits led to syndrome-based detection of STI missed by the sex worker. The final event timeline dataset comprised of 67656 events, 15438 clinic visit events and 52218 peer-outreach visit events.

The key dependent variable of our study is the timing of the first voluntary clinic visit. Of the 2705 sex workers in our sample, 2041 (75.5%) of them voluntarily visited the clinic at least once over their tenure in the project. Thus, the timing of the first voluntary clinic visit is censored for the remaining 664 sex workers. The principal independent variable is the “density of peer educator outreach”, defined as the number of peer educator outreach visits in the 30-day window preceding any given date. Note that our principal independent variable is time-varying, which necessitates the use of an Extended Cox model[27] to allow for time-varying independent variables. We also controlled for the age, client volume, location of sex work (brothel, street, lodge, and home), and the education level (high school or above, some education, illiterate, unknown) of each sex worker. We present several key summary statistics from our dataset in Table 1. We estimated an Extended Cox model using PROC PHREG of the SAS System (Version [9.3] for Windows). Estimated survival curves were produced using the *survival* library in R.

**Table 1:** Descriptive Summary Statistics.

Variables	Mean	Median	S.D.	Min	Max
*Independent variables*					
Total number of peer educator visits	19.30	14.00	18.62	0.00	99
Density of peer educator outreach	0.36	0.29	0.44	-1.99	2.03
Age	29.23	28.00	7.49	18.00	60
Client volume (per month)	6.31	5.00	4.83	1.00	30
Location of sex work Home: 1824 (67.4%) Lodge: 113 (4.2%) Street: 545 (20.2%) Brothel: 223 (8.2%)					
Education level High school or above: 1446 (53.5%) Some education: 37 (1.4%) Illiterate: 1198 (44.3%) Unknown: 24 (0.9%)					
*Dependent variable*					
Duration to first clinic visit (days)p(at least one clinic visit): 75.45%	381.35	232.00	398.51	1.00	1489

## Results

Our first analysis was to examine whether the clinic visits lead to detection of STI over and above that of self-reported STI status by the sex worker. There were a total of 15438 clinic visits across the 2705 sex workers. Of these there were 15097 visits with both self-reported STI-status and clinic-detected STI-symptoms status. There were a total of 5202 (about 34%) visits in which sex workers were detected with at least one of the STI-syndromes; of these, 1941 (about 37%) were instances in which the sex worker came in with either no complaint or a general complaint. This underscores the value of clinic utilization as a key event in the potential detection of STI.

Table 2 presents coefficient estimates from the Extended Cox model. As can be seen, higher density of peer educator outreach is strongly associated with earlier first clinic visit (HR: 1.83, 95% CI, (1.75–1.91, *p* < 0.001). This provides evidence for the effectiveness of peer educator outreach in inducing clinic visit. In terms of other control variables, as expected, we find that sex workers who have a higher education level (High School or Above) tend to visit the clinic earlier (HR: 1.12, 95% CI, 1.03–1.23, *p* < 0.05); we do not find any significant effect with respect to age, client volume, and location of sex work.

**Table 2:** Results from the Extended Cox Model.

Predictor	Parameter Estimate	Standard Error	p-value	Hazard Ratio	95% CI for Hazard Ratio
Density of PE Outreach	0.60	0.02	<.0001	1.83	(1.75, 1.91)
Age	0.00	0.00	0.49	1.00	(0.99, 1.00)
Client volume	-0.01	0.00	0.19	0.99	(0.98, 1.00)
Location of Sex Work					
Home	-0.02	0.08	0.78	0.98	(0.83, 1.15)
Lodge	-0.22	0.14	0.11	0.80	(0.61, 1.05)
Street	-0.02	0.09	0.84	0.98	(0.82, 1.18)
Education level					
High school or above	0.12	0.05	0.01	1.12	(1.03, 1.23)
Some education	-0.39	0.25	0.12	0.68	(0.41, 1.11)
Unknown	-0.21	0.25	0.40	0.81	(0.49, 1.33)

“Brothel” is the reference category for location of sex work; “Illiterate” is the reference category for education level.

**Fig 1:**
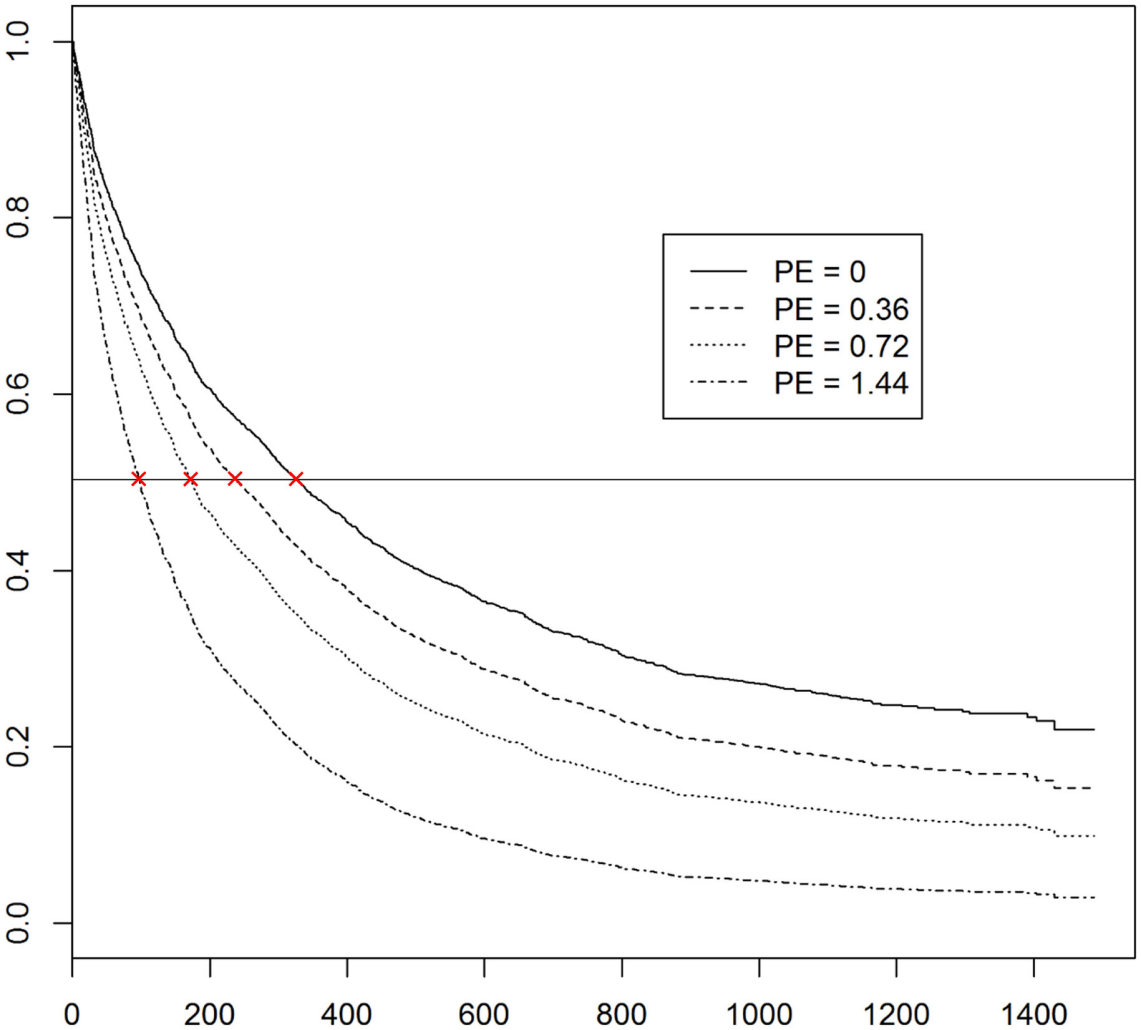
Estimated Survival Curve under different Densities of Peer Educator Outreach. The y-axis represents the event-free survival probability, i.e., likelihood of not experiencing the focal event of first clinic visit. The x-axis represents number of days from first contact. The different lines represent the counterfactuals pertaining to zero, 1X, 2X and 4X the observed outreach density. As the outreach density increases, the event-free survival probability goes down for the same number of days. Alternately, the median time to clinic visit, represented as the cross mark “×” on the horizontal reference line, becomes shorter (moves left) as the outreach density increases from 0 to 1X, to 2X to 4X.

To further quantify the association between peer educator outreach and the timing of the first clinic visit, we compared the estimated survival curve (Fig 1) where the density of peer educator outreach is set to 0 (i.e., no peer educator visit) to the estimated survival curves where the density of peer educator outreach is set to 0.36 (which corresponds to average rate of peer educator visit in our data), 0.72 (doubling the rate of peer educator visits), and 1.44 (quadrupling the rate of peer educator visits), respectively. In each case, the other covariates are set as follows: age is set at 29.23 (the mean value of the dataset), while client volume is set at 6.31 per month (the mean value of the dataset); location of sex work is set at “Home” and education is set at “High School or Above”, the modal value of the dataset (see Table 1).

As can be seen in Fig 1, estimated time to first clinic visits decreases as the density of peer educator outreach becomes higher. The (predicted) median time to first visit when the density of peer educator outreach is set to 0, 0.36, 0.72, and 1.44 is 330, 241, 174, and 98 days, respectively. Thus, compared to no peer educator visits, having 0.36 peer educator visit per month is associated with a reduction of median time to visit by almost three months (89 days), while having 1.44 peer educator visits per month is predicted to reduce median time to first clinic visit by about eight months (232 days). This provides an estimate of the effect size for peer educator outreach, which has implications for staffing of outreach workers. Incidentally, the Avahan common minimum program calls for one peer-outreach visit (to each sex worker) per month, which is around three times the current rate we observed.

### Robustness Checks

To study the robustness of our core result, we conduct a series of robustness checks by specifying a number of alternate models as follows. First, we assess the proportional hazard assumption for the age and client volume variables by including the interaction of age and time (in days) and the interaction of client volume with time (in days) into the Extended Cox model[27]. Both interactions are not statistically significant (*p* > .10), which do not reject the proportional hazard assumption. It is important to note that client volume was modeled as a static control variable because it was collected only once during the registration into the project. It is more likely that client volume varies with time, and should be modeled as a time-varying predictor if such data were available.

Second, we explore the sensitivity of our results with respect to how we operationalize the density of peer educator outreach. Specifically, instead of taking a rolling 30-day average to compute the (time-varying) density of peer educator outreach, we conduct two additional analyses where we take a 15-day average and a 60-day average (respectively) to compute peer educator outreach density per month. Note that, in each case, the metric is scaled so that in each case the independent variable is the density of peer educator outreach in 30 days; in other words, we multiple the 15-day outreach count by 2, whereas we divide the 60-day outreach count by two. The estimated hazard ratio for the model with 15-day average and 60-day average are 1.50 (95% CI, 1.45–1.55, *p* < 0.001) and 2.23 (95% CI, 2.12–2.36, *p* < 0.001), respectively. Thus, our results are fairly robust to the operationalization of density of peer educator outreach.

Third, one potential caveat of our data collection method is that follow-up to sex workers can be lost before the end of the observation window, e.g., if a sex worker relocates to another city, which potentially creates non-random attrition bias. To assess the robustness of our results, we conduct another set of analysis where we artificially censor the record of each sex worker at the date when her last meeting with a peer educator takes place, to put an upper bound on any potential attrition bias. The estimated hazard ratio is 1.39 (95% CI, 1.32–1.46, *p* < 0.001), again showing a positive association between higher density of peer educator outreach and earlier first clinic visit. By comparing this estimate with the estimates presented in Table 2, we find that the hazard ratio is attenuated by around 25%; this suggests that there may be some degree of non-random attrition bias in the data.

Finally, given that our study is observational in nature, our results may be subject to self-selection bias; specifically, it is possible that sex workers who are more willing to meet with a peer educator may also be more willing to visit the health clinic, thus resulting in a spurious correlation between peer educator visit density and likelihood of visiting the clinic. To address this concern, we conducted a robustness check that includes not only the first clinic visit but also the second clinic visit (if any) for each sex worker. We specified an alternate model in which we predicted the likelihood and timing of the second clinic visit using 30-day peer educator visit density in the time interval between the first and second visit. Using this recurrent event survival analysis model[28], the estimated hazard ratio for peer educator outreach is 1.19 (95% CI, 1.16–1.21, *p* < 0.001), which provides further validation for our results. Notice that since every sex worker in the recurrent event analysis had at least one clinic visit, self-selection is not a viable explanation for the effect of peer educator visit density on clinic utilization. The details of this analysis are available from the authors upon request.

## Discussion

We focus on the question of whether peer educator outreach, a key element of many programs aimed at reducing sexually transmitted infections, is associated with earlier clinic visits. Towards that end, we conduct survival analysis using a longitudinal dataset on peer educator outreach and clinic visits from Project *Pragati*, one of the implementation partners of Avahan. Results from an Extended Cox model suggest that higher peer educator outreach density is indeed associated with earlier clinic utilization. Importantly, we also provide an estimate of the magnitude of the effect of peer educator outreach on the timing to first clinic visit, which may have key implications for program staffing decisions. Separately, we find that clinic utilization results in detection of STI-syndromes that the sex worker seems to have missed. This underscores both the importance of peer-led outreach and the role of clinic visits. By encouraging sex workers to come to the clinic, the outreach workers are helping detect STI that would have otherwise gone unnoticed.

These results make the following contribution to the vast scholarship in the research on the role of large scale interventions on STI reduction, including those that assess the cost effectiveness of programs of this scale[29, 30]. The contribution of this research is along the following lines. First, we focus on peer-outreach intensity, a key element of large-scale interventions that has not received much attention at an individual-level. Second, we study timing to clinic utilization, an important event in the course of STI that helps not only treatment for STI, but detection thereof. Third, we take advantage of a fine-grained dataset to specify an Extended Cox model that allows us to model time, include time-varying covariates, and incorporate censoring which may otherwise lead to biased estimates. Fourth, we employ a modeling approach similar to salesforce response models, drawing inspiration from the discipline in marketing to assess the role of peer-educator outreach in what is appropriately viewed as a social marketing or direct marketing intervention as described by the World Bank[31]. Finally, there is a potential programmatic implication from the findings in our study; we observed peer-outreach density of about 0.36 visits per month, which is about a third of the anticipated outreach density of one per month. It is important to investigate why this outreach density is lower than planned, and what might be done to improve it.

Our findings should be interpreted with caution because of a few key limitations. First, due to the observational nature of our data, our results should be interpreted as an association rather than causation. Although a controlled experiment is impractical in this context due to ethical/pragmatic concerns[19], it is important that the public health and research community to use similar panel datasets or other observational study designs to assess the generalizability and validity of the findings reported in this paper. Next, our data come from only Project *Pragati*; the extent to which our findings are generalizable to other outreach efforts is unclear. However, it must be noted that the level of granularity of data needed for developing individual, visit-timing model is not available at other project sites. Another limitation is the analysis presented in this paper does not consider the whether the relationship between peer-educator visit density and clinic visit behavior is mediated by changes to the level of risk of STI perceived by the sex worker or by self-reported STI. We hope to address some of these limitations in future research.

Despite these limitations, we believe that our findings offer some encouraging evidence that peer educator outreach is effective in accelerating the timing of clinic utilization.
